# Psychometric properties of empathy questionnaire for Spanish adolescents

**DOI:** 10.1186/s41155-020-00161-w

**Published:** 2020-10-31

**Authors:** Carlos Salavera, Pablo Usán

**Affiliations:** 1 Research Group OPIICS, University of Zaragoza, Zaragoza, Spain; 2 Faculty of Education, University of Zaragoza, c/ Pedro Cerbuna, 12, 50009 Zaragoza, Spain

**Keywords:** Empathy, Assessment, Adolescents

## Abstract

The aim of this study was to adapt and test the empathy questionnaire in Spanish adolescents (*N =* 701, age *=* 13.47 years). The study involved two different strands: (1) the questionnaire was translated into Spanish, and its internal consistency, factorial structure and convergent validity were assessed; (2) the questionnaire was subject to confirmatory factor analysis.

The results of the confirmatory factor analysis show that the questionnaire’s factors present an aggregate variance of 58.588%, which suggests that the questionnaire is a valid tool to represent affective empathy, cognitive empathy and empathic concern. On the other hand, the confirmatory factor analysis confirmed the sustainability of the model, which comprises three identified factors and twelve items. The empathy questionnaire is easy to understand and can be completed in a short time, so it is considered a useful tool to assess empathy in Spanish adolescents. The results are discussed in the context of theoretical accounts of empathy.

## Background

Empathy is the ability to understand other people’s minds, to feel emotions other than our own and to respond with concern, goodwill and care for other people’s hardship. Generally, it is understood as the comprehension of the emotional state of others (Cohen & Strayer, 1996). Empathy (Vossen, Piotrowski, & Valkenburg, 2015) comprises two interrelated components: affective empathy (the ability to experience other people’s feelings) and cognitive empathy (the ability to understand other people’s feelings). In childhood, low empathy is related to poor relationships with peers, hostility and intimidation. In adolescents, low empathy results in aggression and antisocial behaviour. In adulthood, low empathy leads to child abuse, violence and psychopathy. Conversely, high empathy is related to good social skills, the ability to solve problems and pro-social behaviour; among both children and adults, highly empathetic people have greater chances to share resources, help those in need and care for others (Cuff, Brown, Taylor, & Howat, 2014; Mitsopoulou & Giovazolias, 2015; Williams, O'Driscoll, & Moore, 2014).

### Affective empathy, cognitive empathy and empathic concern

Empathy plays a key role in the development of social behaviour. Initially, empathy was conceptualised from an emotional perspective and was understood as a vicarious emotional response to the emotions perceived in others. Later, it was understood that this one-dimensional approach to empathy erroneously disregarded the role of cognition. Currently, empathy is considered a multi-dimensional concept with both emotional and cognitive components (Vossen et al., 2015).

Based on this, it is important to distinguish between the different components of empathy: (1) Cognitive empathy refers to the ability to adopt the point of view of others and to understand their emotions and feelings. It involves “putting” oneself in the place of others, without judging them from our perspective. For instance, a student understands that one of his classmates likes having a different haircut and dressing in different clothes. (2) Emotional empathy involves feeling what other people feel. For instance, if a student expresses his suffering, his classmates feel it so deeply that they end up crying in response to his ordeal. (3) Empathic or sympathetic concern refers to the ability to understand when someone else needs our help, and this help is offered spontaneously and unconditionally. A student offers his support to a socially isolated classmate to help him overcome this situation.

Research to date has shown that people with high empathy are more prone to develop pro-social and altruistic behaviour (Cuff, Brown, Taylor, & Howat, 2014; Telle & Pfister, 2014), whereas individuals with low empathy tend to be aggressive (Garaigordobil, Martínez, & Aliri, 2013; Gutiérrez, Escartí, & Pascual, 2011; Winter, Spengler, Bermpohl, Singer, & Kanske, 2017).

The aim of this study was to assess the use of the empathy questionnaire (Vossen et al., 2015) in Spanish adolescents, providing Spanish investigators with a hitherto non-existing tool to measure empathy in members of this age group.

Study 1 involved the translation of the questionnaire into Spanish, and the assessment of the questionnaire’s internal consistency, factorial structure and convergent validity. In study 2, the confirmatory factor analysis of the scale was carried out. Following the questionnaire’s theoretical background, the working hypothesis was that the questionnaire follows a three-factor structure, which allows for the assessment of affective empathy, cognitive empathy and empathic concern. The core hypothesis, therefore, was that the questionnaire is a valid tool to measure empathy in Spanish adolescents.

Currently, there are several scales available to measure empathy: the Interpersonal Reactivity Index (IRI; Davis, 1980); the Empathy Index for Children and Adolescents (ACEI; Bryant, 1982); and the Children’s Behavior Questionnaire (CBQ; Rothbart, Ahadi, & Hershey, 1994) with its Empathy Subscale and the Basic Empathy Scale (Jolliffe & Farrington, 2006). Some of these scales do not distinguish between the affective and cognitive component of empathy, considering empathy as a unique construction. In addition, the wording of the elements is sometimes ambiguous. The empathy scale of Vossen et al. (2015) has focused its efforts on guaranteeing to solve these difficulties, ensuring that the elements are clear and unambiguous.

The objective of this study is to develop a validated measure of empathy for Spanish adolescents and to address the aforementioned limitations of the existing scales.

## Method

### Study 1

For the translation of the questionnaire into Spanish, we followed the back-translation method (Muñiz, Elosua, & Hambleton, 2013), which involves the following steps:

1. The original English questionnaire was translated into Spanish by two bilingual persons with experience in the field of psychology. These translations were discussed by the research team, and a first draft was produced.
2. A psychologist with work experience in English-speaking countries assessed the conceptual equivalence, clarity and intuitiveness of the expressions and answer contained in the first draft, suggesting corrections that led to the second draft.
3. This second draft was presented to experts in the field of empathy.
4. The second Spanish draft was back-translated into English by a bilingual native-English speaker professional translator to analyse and verify the correspondence of both.
5. A pilot test with 50 respondents was carried out to assess comprehensibility, response time, clarity of questions and suitability of answers. No changes in the content of the questionnaire were necessary.
6. Following this test, the research team developed a third Spanish version of the questionnaire which was to be presented to a sample of Spanish adolescents.

### Study 2

Using the AMOS v.24 statistical program, a structural equation model was developed to validate and quantify the causal relationships between the questionnaire’s items and questions. This technique combines factor analysis with linear regression to test the degree of fit of observed data to a hypothesised model and expressed by means of a path diagram. As a result, it provides the values belonging to each relationship and, more importantly, a statistic that expresses the degree to which the data fit the proposed model, confirming its validity. Following Batista and Coenders (2000) and Rhemtulla, Brosseau-Liard, and Savalei (2012) based on the number of variables, we used the maximum likelihood estimation method, rather than the weighted least squares method.

### Participants

The population of samples included 701 subjects: 343 men (48.93%) and 358 women (51.07%); the average age of participants was 13.47, ranging from 12 to 18, with a standard deviation of 1.796. The first study included 337 respondents (166 men and 171 women) and the second 364 respondents (177 men and 187 women). Both samples were homogeneous. All respondents were volunteers, and parents and children signed an informed consent form; all the guidelines established by the Declaration of Helsinki and ethical criteria for research with humans were followed. The answers were treated anonymously. Representativeness estimates yielded a 95% confidence level and a 5% sampling error, and it was concluded that the final results are representative of the province of Zaragoza. The study was designed as an instrumental study (Ato, López, & Benavente, 2013).

### Instruments

#### Empathy questionnaire (Vossen et al., 2015)

It is a 12-item scale that consists of three dimensions with four items each: cognitive empathy, affective empathy and empathic concern. The respondents must read a statement and answer on a 5-point Likert scale divided as follows: (1) strongly disagree, (2) disagree, (3) neutral, (4) agree and (5) strongly agree. Four items were generated to measure affective empathy (e.g. “When a friend is scared, I feel afraid”), 6 were generated to measure cognitive empathy (e.g. “I can often understand how people are feeling even before they tell me”) and 4 were generated to measure sympathy (e.g. “I feel sorry for someone who is treated unfairly”). Permission was sought from the authors of questionnaire to use it for research purposes. The questionnaire was translated into Spanish following the back-translation method (Muñiz et al., 2013).

#### Interpersonal Reactivity Index (Davis, 1980)

For the study, the Spanish version of IRI was used (Pérez-Albéniz, de Paúl, Etxeberría, Montes, & Torres, 2003).

IRI is a self-report questionnaire used to measure empathy, comprising 28 statements and 5-point Likert scale responses ranging from 0 (it does not describe me well) to 4 (it describes me very well). The questionnaire is divided into four independent dimensions, with seven items each: (1) fantasy (tendency of subjects to identify with fictional characters, such as book and movie characters), (2) perspective (tendency or ability of subjects to adopt the perspective or point of view of others), (3) empathic concern (feelings of sympathy, compassion and concern for others) and (4) personal anguish (feelings of anxiety and discomfort caused by the negative experiences suffered by others). For this study, the perspective and empathic concern dimensions were selected, which are often used to measure the affective and cognitive components of empathy, respectively.

### Procedure

In order to recruit the participants, schools were contacted by phone, and after they agreed to participate, a list of centres was drafted. When the questionnaires were handed out, the participants were informed about the aim of the study, and the importance of all items being answered was stressed, in addition to the minimum task instructions, and was highlighted, as this could influence their responses to the questionnaires. The respondents, who read the questionnaires silently, were given 45 min to complete the questionnaires and sign the informed consent form. Every time, it was emphasised that the information provided was anonymous and confidential. All participants, in addition to parents in the case of minors (in Spain, minors are up to 18 years old), signed the informed consent form. The data was collected between October and November 2018.

### Analysis of the data

The statistical analysis of the data was carried out with the SPSS v.26.0 program. The data was initially subject to normality and variance tests, and the use of parametric techniques was selected for further analysis. In all cases, we worked at the lowest possible level of significance. Bilateral tests were performed. For two-group hypothesis testing, we used Student’s *t* distribution. Cronbach’s alpha was used to test the reliability of the scale. The structure of the scale was tested by means of an exploratory factor analysis (EFA) to explore the factorial structure of the data. Since the data were normally distributed and the factors were expected to be correlated, a principal component analysis was used with a varimax rotation method.

Finally, confirmatory factor analysis was carried out with a model of structural equations, using the AMOS v.24 statistical program.

## Results

The first study included 337 respondents (166 men and 171 women). Empathy scores (Table 1) were higher in women in all three factors under analysis (cognitive empathy, affective empathy and empathic concern), with near-average scores (Cohen’s *d*) in all variables.

**Table 1:** Empathy questionnaire descriptive items

*Factors*	*M(SD), males*	*M(SD), females*	*Cohen’s d*
*Affective empathy*	12.90 (2.77)	13.88 (2.48)	0.37
*Cognitive empathy*	10.56 (3.28)	11.86 (3.12)	0.40
*Empathic concern*	15.30 (3.05)	16.66 (2.76)	0.46

The results were organised according to the different stages in our research programme. The results of study 1 included construct validity, internal consistency analysis, convergence analysis and exploratory factor analysis (EFA), and those of study 2 included confirmatory factor analysis (CFA).

### Study 1

#### Construct validity

The aim of this step is to validate the empathy questionnaire (Vossen et al., 2015), following its translation.

After testing the viability of factor analysis, we used a principal component analysis with a varimax rotation: the correlation matrix showed a high number of correlations (87.9%) with a value over .30 (coefficient determination of .002), while Bartlett’s test of sphericity showed that the variables were not independent (Bartlett test = 2870.823, *p* < .001). The Kaiser-Meyer Adequacy (KMO) test yielded a result of .870, which suggests that pairwise variable correlations are nearly entirely explained by the other variables. All the values yielded by the Measures of Sampling Adequacy (MSA) test were above .80. These results show the suitability of the correlation matrix for factor analysis. As shown in Table 2, after assigning an item to all factors with a load factor of .40 or more, three values with an eigenvalue of 1 or above were obtained, explaining 58.588% of total variance. In this section, we would highlight the good factor load obtained. The greatest saturation was associated to affective empathy (26.352%), followed by factor of cognitive empathy (22.066%), and empathic concern (10.171%).

**Table 2:** Standardised factors of the empathy questionnaire

		Components		
		Cognitive empathy	Affective empathy	Empathic concern
Factor 1: cognitive empathy				
1.	I can often understand how people are feeling even before they tell me/A menudo puedo entender cómo se siente la gente incluso antes de que me lo digan.	.767		
2.	I can tell when a friend is angry even if he/she tries to hide it/Puedo decir cuando un amigo está enfadado incluso si él/ella trata de ocultarlo.	.723		
3.	I can tell when someone acts happy, when they actually are not/Puedo decir cuando alguien actúa como si estuviera feliz, cuando en realidad no lo está.	.775		
4.	I can easily tell how others are feeling/Puedo decir fácilmente cómo se sienten los demás.	.759		
Factor 2: affective empathy				
5.	When a friend is scared, I feel afraid/Cuando un amigo tiene miedo, tengo miedo.		.748	
6.	When my friend is sad, I become sad too/Cuando mi amigo está triste, yo también me pongo triste.		.701	
7.	When a friend is angry, I feel angry too/Cuando un amigo está enfadado, yo también me enfado.		.816	
8.	When people around me are nervous, I become nervous too/Cuando las personas a mi alrededor están nerviosas, yo también me pongo nervioso.		.683	
Factor 3: empathic concern				
9.	I feel sorry for someone who is treated unfairly/Siento pena por alguien que es tratado injustamente.			.784
10.	I feel concerned for other people who are sick/Me preocupo por otras personas que están enfermas.			.717
11.	I am concerned for animals that are hurt/Me preocupan los animales heridos.			.685
12.	I feel sorry for a friend who feels sad/Siento pena por un amigo que se siente triste.			.706
Variance percentage		22.066%	26.352%	10.171%

Factors with a load value under .30 are not shown on the table

Table 2 shows the matrix of components resulting from the principal component analysis. The table shows the factors and their associated questionnaire items, as well as the saturation values. For comparison purposes, the data model was considered adequate if the chi-square and degrees of freedom values were 3 or below (Hu & Bentler, 1999). Our scales yielded values below 3, showing that they are well adjusted and are internally consistent.

#### Convergence analysis

The correlations yielded by the empathy questionnaire were compared with those obtained with the IRI’s perspective taking and empathic concern values (Table 3). The dimensions of the empathy questionnaire showed statistically significant correlations with the other variables. The correlations with personality factors were negative, whereas concerning the two IRI scales, affective empathy yielded lower results than cognitive empathy and empathic concern.

**Table 3:** Correlations with dispositional empathy (IRI)

	Cognitive empathy	Affective empathy	Empathic concern
IRI-perspective taking	.31**	.09*	.33**
IRI-empathic concern	.23**	.20**	.50**

Note: * *p* > 0.01, ** *p* < 0.001

#### Reliability

In order to test the reliability of the questionnaire, Cronbach’s coefficient was used to calculate internal consistency, because this test has not been translated into Spanish, and we wanted to test our version. The results to all of the questionnaire’s items yielded α results (Table 4) of .80 or above, and it can thus be assumed that the items measure the same construct and are highly correlated. In terms of reliability, the questionnaire yielded a high score with .872. Cronbach’s alpha coefficients of the total scale (total = .872; cognitive empathy = .786; affective empathy = .744; empathic concern = .750) were moderately high, which shows that the different items in the questionnaire are highly consistent.

**Table 4:** Internal consistency of the factors in the empathy questionnaire

		Mean of the scale if the item is eliminated	Variance of the scale if the item is eliminated	Corrected element-total correlation	Cronbach’s alpha if the item is eliminated
Factor 1: cognitive empathy					
1.	I can often understand how people are feeling even before they tell me.	37.793	40.897	.522	.826
2.	I can tell when a friend is angry even if he/she tries to hide it.	37.520	41.405	.465	.830
3.	I can tell when someone acts happy, when they actually are not.	38.081	41.153	.441	.831
4.	I can easily tell how others are feeling.	38.115	40.970	.443	.831
Factor 2: affective empathy					
5.	When a friend is scared, I feel afraid.	38.734	38.943	.559	.823
6.	When my friend is sad, I become sad too.	37.859	38.195	.601	.819
7.	When a friend is angry, I feel angry too.	38.754	38.942	.545	.824
8.	When people around me are nervous, I become nervous too.	38.298	39.108	.435	.834
Factor 3: empathic concern					
9.	I feel sorry for someone who is treated unfairly.	36.966	40.418	.530	.825
10.	I feel concerned for other people who are sick.	37.161	39.690	.573	.822
11.	I am concerned for animals that are hurt.	37.474	39.551	.410	.836
12.	I feel sorry for a friend who feels sad.	37.254	39.182	.563	.822

### Study 2

#### Confirmatory factor analysis

Confirmatory factor analysis was used to test the internal structure of the data; this is the most appropriate statistical framework to evaluate the validity and reliability of each item, rather than the overall data, allowing the researcher to design and adapt the questionnaire (Batista & Coenders, 2000).

**Fig. 1:**
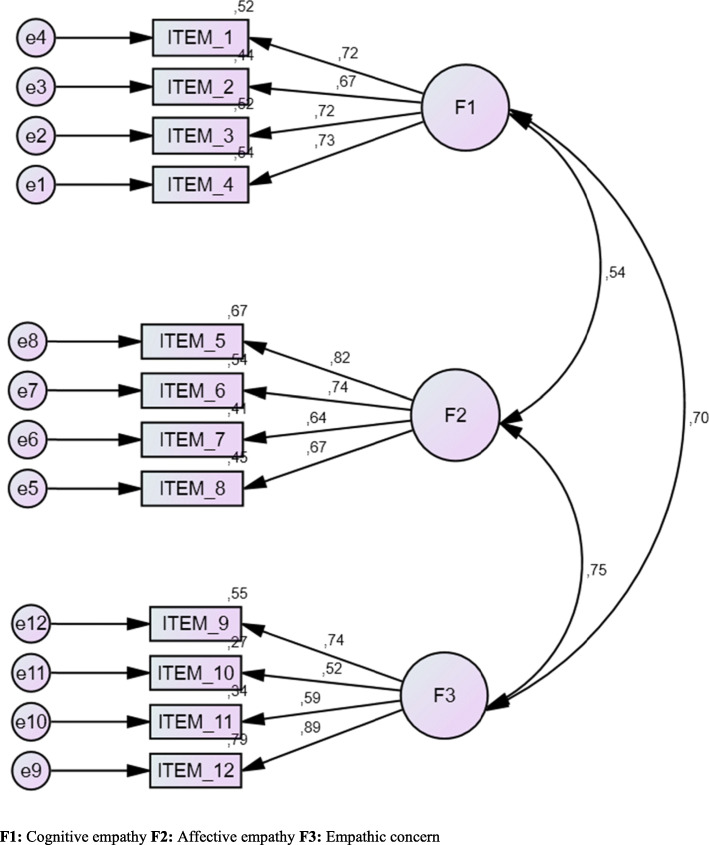
Standardised solution of the CFA of the empathy scale

Figure 1 shows the result of the CFA of the model developed in the exploratory study. This was carried out with structural equations following a maximum likelihood model. The results confirmed the validity of the model, since the results presented a sustainable model constituted by the three identified factors and a total of 12 items.

Concerning the adjustment of the model, the adjustment indexes were adequate, which confirms that the model proposed for the factor structure is sustainable: *χ*^2^(51) = 85.659; *p* < .001; *χ*^2^/gl = 1.679; CFI = .986; NFI = .969; TLI = .981; RMSEA = .032, 95% CI (.021–.044). The confirmatory factor analysis used a three-factor model. This is the a priori structure, which confirms that the results of the model are fully confirmatory. The results suggest that the model is reasonably well adjusted. These results (Fig. 1) indicate that the model is optimally adjusted to the data and that the questionnaire is thus a valid tool.

Subsequently, the different factor components were analysed in order to ascertain the existence of significant correlations between the different dimensions: cognitive empathy, affective empathy and empathic concern. The correlations were as follows: *r* = .54 between cognitive empathy and affective empathy, *r* = .70 between cognitive empathy and empathic concern and *r* = .75 between affective empathy and empathic concern (*p* < .001; see Fig. 1).

Figure 1 shows the hierarchical model of the three factors.

## Discussion

The aim of this study was to test the use of the empathy questionnaire to assess Spanish teenagers. The results confirm the validity of the questionnaire, which was subject to a strict translation process and to the analysis of its psychometric features. This process aimed to guarantee that the translated questionnaire was culturally and linguistically compatible with the original English version, as well as to ensure its internal consistency, validity and factorial structure.

The main results of the factor analysis indicate a three-factor structure and a high saturation value for all items, reflecting the internal consistency of the questionnaire (these results are similar to those yielded by the original questionnaire and previous translations) (Vossen et al., 2015; Wang, Wen, Fu, & Zeng, 2017; Zengin, Yalnizoglu-Çaka, & Çinar, 2018).

These results show that the factors in the empathy scale present an aggregate variance of 58.588%. Results provide evidence for validity also in connection with other variables, showing that the different elements of the scale are highly consistent, which emphasises the model’s reliability. Also, the exploration of the underlying factorial structure has shown that the reliability indexes apply to all three subscales, with results that mirror those obtained with the original scale. It can, therefore, be argued that the translation of the scale into Spanish does not undermine its value as a tool. The structure has been validated on three levels, which provides solid empirical evidence for the validity of the model. In addition, the factors of the scale were highly correlated with dispositional empathy factors, especially empathic concern.

This constitutes preliminary evidence for links between empathy questionnaire and IRI (personal reactivity index), which also emphasises the validity of the questionnaire, a tool which is both easy to apply and suitable for the purposes for which it was designed. One of the distinguishing features of this scale rests with its theoretical foundations, according to which empathy plays a crucial role in the way people deal with their emotions and the way they react according to capacity, motivation and context.

The model proposed for the factorial structure is sustainable, with three identified factors and twelve items overall. We think that these results show that the structure is solid, and reveal that the Spanish translation faithfully replicates the original theoretical structure.

The data yielded by the study with Spanish adolescents is satisfactory, fitting the underlying theoretical model and showing high internal consistency and validity. The study has also provided useful data concerning affective empathy, cognitive empathy and empathic concern in adolescents. These are key features for keeping good interpersonal relationships, and for efficiently appraising emotions and adjusting social and emotional conducts accordingly. Therefore, consideration should be given to applying instruments that assess interpersonal relationships and emotional regulation so that it is possible to observe the correlations between these and empathy. Furthermore, the relationship between empathy and affective communication capacity, respect for others, pro-social attitudes, emotions, etc. should be studied (Batchelder, Brosnan, & Ashwin, 2017; Belacchi & Farina, 2012; Bisquerra, 2009; Cook et al., 2008; Gini et al, 2007; Kinnaman & Bellack, 2012; Kotsou, Leys, & Fossion, 2018; Lockwood et al, 2014; Salavera, Usán and Jarie, 2017; Salavera et al, 2020; Thompson, 2000, Wai & Tiliopoulos, 2012), what will benefit from the future use of the empathy scale in relation to these variables, as well as from deeper analyses and even a revaluation of the variables.

These results must be interpreted taking the limitations of the study into account. Although the sample is statistically relevant, the questionnaire should be applied to other population groups, in which the correlation of empathy and other variables may be greater. Lateral studies are also advisable to evaluate the evolution of empathy over time. In addition, empathy often appears in relation with other skills, for instance cognitive skills, and this relationship is worth exploring. Similar studies could also target special groups, such as adolescents with social anxiety or autism or with different empathy characteristics.

## Conclusions

The main conclusion of the study is that the empathy questionnaire is a valid tool to assess empathy in adolescents. The questionnaire has shown to be a valuable psychometric tool, with high values of factorial validity and internal consistency; in addition, it is quick and easy to use. Although more research is needed, our results show that the empathy questionnaire is a valid tool to measure empathy in adolescents.

## Data Availability

The authors do not wish to make the data available as it contains information that could identify specific individuals.

## Abbreviations

AMES: Adolescent Measure of Empathy and Sympathy
CFA: Confirmatory factor analysis
EFA: Exploratory factor analysis
IRI: Interpersonal Reactivity Index
KMO: Kaiser-Meyer Adequacy
MSA: Measures of Sampling Adequacy
